# Time-Dependent Diffusion MRI-Based Microstructural Mapping for Characterization of Cribriform and Intraductal Carcinoma Morphologies in Prostate Cancer: A Preliminary Study

**DOI:** 10.3390/cancers18132056

**Published:** 2026-06-25

**Authors:** Yanchun Wei, Shicong Yang, Tuo Ren, Zhihua Wen, Xiang Li, Jian Ling, Jinhua Lin, Yan Guo, Xueying Zhao, Huanjun Wang, Yanling Chen

**Affiliations:** 1 Department of Radiology, The First Affiliated Hospital, Sun Yat-sen University, Guangzhou 510080, China; weiych26@mail2.sysu.edu.cn (Y.W.);; 2 Department of Pathology, The First Affiliated Hospital, Sun Yat-sen University, Guangzhou 510700, China; yangshc@mail.sysu.edu.cn; 3 Department of Medical Ultrasound, Division of Interventional Ultrasound, The First Affiliated Hospital, Sun Yat-sen University, Guangzhou 510080, China; 4 MR Research, GE Healthcare, Beijing 100093, China

**Keywords:** prostate cancer, cribriform, intraductal carcinoma, time-dependent diffusion magnetic resonance imaging

## Abstract

**Background:** Intraductal carcinoma (IDC) and invasive cribriform (Cr) histologic patterns are important adverse morphologies in prostate cancer (PCa) and may influence pretreatment risk stratification. This study evaluated the feasibility of time-dependent diffusion magnetic resonance imaging (t_d_-dMRI)-based microstructural mapping for preoperative characterization of these aggressive morphologies. **Methods:** This retrospective study included 95 men with pathologically confirmed PCa on radical prostatectomy specimens from March 2023 to March 2025. T_d_-dMRI was performed using pulsed and oscillating gradient diffusion sequences. Microstructural parameters, including extracellular diffusivity (D_ex_), cell diameter (d), intracellular volume fraction (f_in_), cellularity, and diffusivities at 0, 17, and 33 Hz (ADC_0Hz_, ADC_17Hz_, and ADC_33Hz_), were estimated using a two-compartment model. Conventional apparent diffusion coefficient (ADC_DWI_) values were obtained from standard diffusion-weighted imaging. Parameters were compared between tumors with and without Cr/IDC patterns, and diagnostic performance was assessed using receiver operating characteristic analysis. Pairwise comparisons of AUCs were performed using the DeLong test. **Results:** Among 95 participants, 62 (65.3%) had Cr/IDC patterns. Compared with Cr/IDC-negative tumors, Cr/IDC-positive tumors showed higher f_in_ and cellularity (both *p* < 0.001) and lower ADC_DWI_, ADC_0Hz_, ADC_17Hz_, and ADC_33Hz_ values (all *p* < 0.05). D_ex_ and d did not differ significantly between groups. Among t_d_-dMRI-derived parameters, f_in_ showed the highest diagnostic performance (AUC = 0.757; 95% CI, 0.654–0.860). **Conclusions:** T_d_-dMRI-based microstructural mapping demonstrates promise for characterizing the Cr/IDC morphologies in PCa.

## Simple Summary

Cribriform and intraductal carcinoma morphologies are important adverse histologic patterns in prostate cancer and require more intensive management. However, these adverse histologic patterns are difficult to identify before surgery using conventional imaging alone. This study explored whether time-dependent diffusion magnetic resonance imaging could provide microstructural information related to these morphologies. Tumors with cribriform or intraductal carcinoma showed higher intracellular volume fraction and cellularity, suggesting denser tumor structure. Intracellular volume fraction showed favorable performance for differentiating these patterns and remained associated with Cr/IDC status after adjustment for Gleason grade. These findings suggest that time-dependent diffusion magnetic resonance imaging may provide biologically relevant information regarding prostate cancer aggressiveness, although further prospective studies are required to validate its clinical utility.

## 1. Introduction

Prostate cancer (PCa) is the fourth most commonly diagnosed malignancy and the eighth leading cause of cancer-related death worldwide [1]. Although Gleason score remains a key indicator of tumor aggressiveness, the prognostic significance of cribriform growth pattern (Cr) and intraductal carcinoma (IDC) has been increasingly recognized [2,3]. These high-risk morphologies are linked to genomic instability, biochemical recurrence, and distant metastasis; accordingly, current PCa reporting guidelines recommend documenting these patterns in biopsy and prostatectomy specimens [4,5]. The presence of Cr/IDC in prostate biopsy specimens is regarded as a contraindication to active surveillance and minimally invasive focal therapy [6,7]. In addition, detection of Cr/IDC enhances pretreatment risk stratification using the Cancer of the Prostate Risk Assessment and National Comprehensive Cancer Network frameworks [8]. Therefore, early identification of these aggressive subtypes is crucial for clinical decision-making and prognosis prediction.

Although multiparametric magnetic resonance imaging (mpMRI), especially diffusion-weighted imaging, plays an indispensable role in PCa detection, it remains limited for characterizing cellular microstructure within tumors. Advances in multidimensional diffusion magnetic resonance imaging, including different diffusion weightings (q-space) and varying diffusion times (t-space), have expanded the ability to probe tissue microstructure and broadened the scope of PCa research [9,10,11]. In particular, time-dependent diffusion magnetic resonance imaging (t_d_-dMRI) enables characterization of cellular architecture by capturing restricted water diffusion across diffusion times and relating diffusion-time dependence to specific microstructural parameters [12,13,14]. Prior studies have indicated that t_d_-dMRI-derived parameters can discriminate PCa across Gleason scores and correlate with pathologic findings [15]. However, its feasibility for describing Cr/IDC morphologies in PCa has not yet been explored.

Cribriform architecture refers to solid proliferation of PCa cells forming multiple glandular lumina without intervening stroma, whereas IDC involves malignant cells filling and distending preexisting ductal or glandular spaces [16]. Although Cr and IDC are distinct pathologic entities, they frequently coexist and are often grouped together clinically because of overlapping morphology and similarly poor prognosis [17,18,19,20]. Because tumors with Cr/IDC morphologies exhibit distinct histologic architecture, we hypothesized that t_d_-dMRI-based microstructural mapping may add value for noninvasive detection of these high-risk patterns. This study aimed to evaluate the clinical utility of t_d_-dMRI-based microstructural mapping for noninvasive characterization of Cr/IDC patterns in PCa.

## 2. Materials and Methods

### 2.1. Study Participants

**Figure 1:**
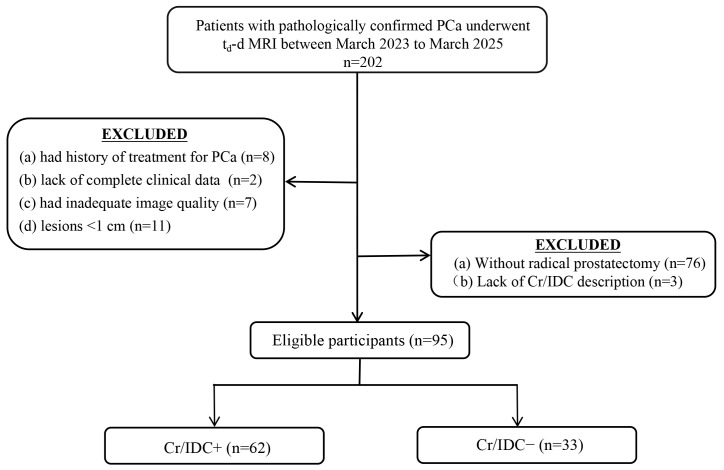
Flowchart showing participant enrollment. Abbreviations: Cr/IDC, cribriform/intraductal carcinoma histologic pattern; PCa, prostate cancer.

This retrospective study included consecutive patients with pathologically confirmed PCa who underwent preoperative multiparametric MRI including t_d_-dMRI at our institution between March 2023 and March 2025. The source population was limited to surgically treated patients with available prostatectomy specimens as the histopathologic reference standard. Patients were excluded because of prior treatment for PCa, incomplete clinical information, insufficient image quality, lesions smaller than 1 cm that were unsuitable for reliable region-of-interest (ROI) analysis, absence of radical prostatectomy pathology, or lack of Cr/IDC description in the pathology report. The patient selection flowchart is shown in Figure 1.

The study was approved by the ethics committee of the First Affiliated Hospital, Sun Yat-sen University [(2023)094]. Written informed consent was obtained from all participants.

### 2.2. Image Acquisition

Prostate magnetic resonance imaging was performed on a 3.0-T scanner (Premier, GE Healthcare, Waukesha, WI, USA) with a nominal maximum gradient amplitude of 80 mT/m and a maximum slew rate of 200 T/m/s, using a 30-channel AIR coil. The protocol included t_d_-dMRI and standard-of-care mpMRI, consisting of axial T1-weighted fast spin-echo; T2-weighted fast spin-echo in axial, coronal, and sagittal planes; echo-planar diffusion-weighted imaging with b values of 50, 1000, and 1500 s/mm^2^; and dynamic contrast-enhanced MRI. Detailed imaging parameters are listed in Table S1.

T_d_-dMRI included an oscillating gradient spin-echo (OGSE) sequence with a trapezoid-cosine waveform and a pulsed gradient spin-echo (PGSE) sequence. OGSE data were acquired at 33 Hz (effective diffusion time = 7.6 ms; two cycles; b = 300 and 600 s/mm^2^) and 17 Hz (effective diffusion time = 14.8 ms; one cycle; b = 200, 400, 800, and 1200 s/mm^2^). PGSE data were acquired with diffusion-gradient duration δ = 8 ms and separation Δ = 70 ms (equivalent frequency = 0 Hz; effective diffusion time = 67.3 ms; b = 200, 400, 800, and 1200 s/mm^2^). The following parameters were identical for both sequences: repetition time = 7000 ms; echo time = 150 ms; diffusion directions = 6; field of view = 230 × 230 mm; in-plane resolution = 2.7 × 2.7 mm; matrix = 86 × 86; slices = 10; and slice thickness = 5 mm. The total acquisition time for the t_d_-dMRI protocol was approximately 7 min 40 s. Detailed diffusion encoding parameters for OGSE and PGSE are provided in Table S2.

### 2.3. Image Processing

T_d_-dMRI data were fitted using the Imaging Microstructural Parameters Using Limited Spectrally Edited Diffusion (IMPULSED) model, a two-compartment model comprising intracellular and extracellular components [21]: S = f_in_·S_in_(d,D_in_,b) + (1 − f_in_)·S_ex_, where S is the measured diffusion-weighted magnetic resonance imaging signal; S_in_ and S_ex_ are the intracellular and extracellular signals, respectively; f_in_ is the intracellular volume fraction; d is the mean cell diameter; b is the diffusion-weighting factor determined by the diffusion gradients; and D_in_ is the intracellular diffusivity. S_in_ was modeled using analytical expressions [22] for diffusion in spheres under trapezoidal OGSE encoding. D_in_ was fixed at 1.0 μm^2^/ms to stabilize fitting [15,23]. S_ex_ was modeled as S_ex_ = exp(−b·D_ex_), with extracellular diffusivity (D_ex_) assumed to be independent of diffusion time [21,24,25]. Microstructural parameters, including D_ex_, d, and f_in_, were estimated by least-squares curve fitting using MATLAB 2018a (MathWorks, Natick, MA, USA). Parameter bounds were 0 < d < 30 μm, 0 < f_in_ < 1, and 0 < D_ex_ < 3.5 μm^2^/ms. To avoid local minima, fitting was repeated with 100 randomized initializations, and the solution with the smallest residual was retained. Cellularity was calculated as (f_in_/d) × 100. ADC at each diffusion time was estimated from S/S0 = exp(−b·D) by log-linear fitting to yield ADC_0Hz_ (PGSE, b = 800 s/mm^2^), ADC_17Hz_ (OGSE, b = 800 s/mm^2^), and ADC_33Hz_ (OGSE, b = 600 s/mm^2^).

For MRI-pathology co-registration, two experienced radiologists (Y.G. and J.L., with 30 and 20 years of prostate imaging experience, respectively) and a genitourinary pathologist (S.C.Y., with 20 years of experience) reviewed the corresponding MRI and pathology slides for each patient and identified the dominant tumor lesion. Tumor ROIs were manually delineated on each MRI slice for the entire lesion with reference to T2-weighted images and DWI by two additional radiology fellows (Y.L.C. and Y.C.W., each with 3 years of prostate imaging experience), who were blinded to clinical information except lesion location. Lesion edges, artifacts, necrosis, hemorrhage, and calcification were carefully excluded during ROI delineation. Fitted microstructural parameters (D_ex_, d, f_in_, cellularity, ADC_0Hz_, ADC_17Hz_, and ADC_33Hz_) were averaged across corresponding ROIs. In addition, the conventional ADC value were also calculated from the standard DWI(ADC_DWI_) to test the added value of t_d_-dMRI over the standard-of-care mpMRI.

### 2.4. Histopathologic Analysis

The presence or absence of cribriform architecture and intraductal carcinoma was assessed on radical prostatectomy specimens according to International Society of Urological Pathology (ISUP) criteria [26]. Cribriform architecture was defined as confluent epithelial proliferation forming multiple gland-like lumina without intervening stroma. Intraductal carcinoma was defined as malignant epithelial cells filling and expanding preexisting prostatic ducts or glands. Cr/IDC status was assessed at the patient/dominant-lesion level. A tumor was classified as Cr/IDC-positive if either cribriform architecture or intraductal carcinoma was identified in the corresponding prostatectomy specimen. The percentage of Cr/IDC components was not routinely quantified. Additional recorded pathologic features included Gleason score and International Society of Urological Pathology grade group (2014 recommendations), pathologic T stage (AJCC TNM, 8th edition), D’Amico risk group, and aggressive pathologic features: extraprostatic extension (EPE), seminal vesicle invasion (SVI), perineural invasion (PNI), lymphovascular invasion (LVI), and positive surgical margin (PSM). These histologic characteristics were determined based on prostatectomy specimens.

### 2.5. Statistical Analysis

Continuous variables were compared using Student’s *t* test or the Mann–Whitney U test depending on data distribution. Normality was assessed by the Shapiro–Wilk test. Categorical variables were compared using the Pearson chi-square test or Fisher’s exact test. Sensitivity analyses adjusted for Gleason grade were performed to evaluate associations between Cr/IDC status and each t_d_-dMRI-derived parameter using multivariable linear regression, with adjusted *p*-values reflecting the effect of Cr/IDC patterns after controlling for tumor grade. Diagnostic performance of each microstructural parameter was assessed using receiver operating characteristic (ROC) analysis, with sensitivity, specificity, and accuracy calculated at the optimal threshold based on the Youden index. Areas under the ROC curves (AUCs) were compared using the DeLong test, and internal validation was performed using 1000-sample bootstrap resampling. To explore the independent predictors for Cr/IDC patterns, supplementary univariate and multivariable logistic regression analyses were conducted including preoperative clinical variables, conventional MRI metrics, and t_d_-dMRI parameters. Multicollinearity was assessed using variance inflation factors (VIF), and variables with statistical significance in univariate analysis were retained for multivariable modeling after careful consideration of collinearity. If sufficient independent predictors were identified, a combined diagnostic model will be constructed and evaluated for diagnostic performance.

Interobserver agreement was evaluated using the intraclass correlation coefficient (ICC), interpreted as excellent (≥0.80), good (0.60–0.79), fair (0.40–0.59), or poor (<0.40). Statistical analyses were performed using GraphPad Prism (version 10.1.2; GraphPad Software, San Diego, CA, USA) and R software (version 4.1.1; R Foundation for Statistical Computing, Vienna, Austria). *p* < 0.05 was considered statistically significant.

## 3. Results

### 3.1. Patient Characteristics

In total, 95 participants (median age, 68 years; interquartile range, 62–72 years) were included; 62 (65.3%) had Cr/IDC-positive PCa and 33 (34.7%) had Cr/IDC-negative PCa. Prostate-specific antigen (PSA) and prostate-specific antigen density (PSAD) levels differed between groups [PSA, 13.28 (9.98, 26.26) vs. 8.62 (5.64, 13.96) ng/mL; *p* = 0.003; PSAD, 0.43 (0.25, 0.72) vs. 0.26 (0.15, 0.41) ng/mL^2^; *p* = 0.001]. Baseline participant characteristics are summarized in Table 1.

### 3.2. Pathologic Features of Tumors with Cr/IDC Architecture

There was a significant difference in histologic grade between tumors with and without Cr/IDC architecture. Cr/IDC-positive tumors were associated with higher ISUP grade group (*p* < 0.001). PNI and PSM were more commonly found in PCa with Cr/IDC architecture (*p* = 0.039 and *p* = 0.007, respectively), whereas no significant differences were observed for EPE, SVI and LVI (all *p* > 0.05). Furthermore, Cr/IDC-positive tumors were more frequently classified into higher D’Amico risk groups (*p* < 0.001). Pathologic characteristics are summarized in Table 2.

### 3.3. Comparison of Microstructural Features Between Cr/IDC-Positive and Cr/IDC-Negative Tumors

**Figure 2:**
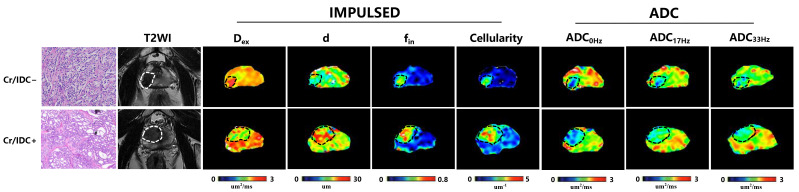
Representative microstructural maps of Cr/IDC-positive and Cr/IDC-negative PCa. The while/black dash contours indicate cancerous regions based on manual delineation. Corresponding pathologic findings based on H&E staining and the T2-weighted images at similar axial locations are shown in the first and second columns, followed by the IMPULSED-derived maps (D_ex_, d, f_in_, and cellularity) and diffusivity maps (ADC_0Hz_,ADC_17Hz_ and ADC_33Hz_). Abbreviations: Cr/IDC, cribriform/intraductal carcinoma histologic pattern; D_ex_, extracellular diffusivity; d, cell diameter; f_in_, intracellular volume fraction; ADC, apparent diffusion coefficient; IMPULSED, Imaging Microstructural Parameters Using Limited Spectrally Edited Diffusion.

Figure 2 shows IMPULSED-fitted microstructural parameter maps for PCa with and without Cr/IDC patterns, together with ADC maps acquired at different oscillating frequencies. Within the tumor regions (dashed contours), f_in_ and cellularity were higher in Cr/IDC-positive tumors, whereas diffusivity measurements at 0, 17, and 33 Hz were lower.

**Figure 3:**
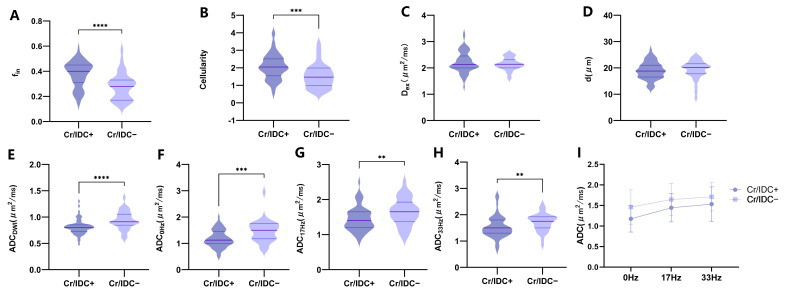
Violin plots comparing microstructural parameters between Cr/IDC-positive and Cr/IDC-negative PCa. (**A**) f_in_, (**B**) cellularity, (**C**) D_ex_, (**D**) d, (**E**) ADC_DWI,_ (**F**) ADC_0Hz_, (**G**) ADC_17Hz_, (**H**) ADC_33Hz_, and (**I**) Diffusion time-dependent changes in ADC with oscillating frequency. Abbreviations: ADC_DWI_, ADC derived from diffusion-weighted imaging; ADC_0Hz_, diffusivity at 0 Hz; ADC_17Hz_, diffusivity at 17 Hz; ADC_33Hz_, diffusivity at 33 Hz; Cr/IDC, cribriform/intraductal carcinoma histologic pattern; D_ex_, extracellular diffusivity; f_in_, intracellular volume fraction. ** *p* < 0.01; *** *p* < 0.001; **** *p* < 0.0001. *p* ≥ 0.05 is not denoted in the plots.

Quantitatively, f_in_ and cellularity were significantly higher in the Cr/IDC-positive group (both *p* < 0.001; Figure 3A,B). No significant differences were observed for D_ex_ or d (*p* = 0.628 and *p* = 0.439, respectively; Figure 3C,D). ADC_DWI_, ADC_0Hz_, ADC_17Hz_, and ADC_33Hz_ values were significantly lower in the Cr/IDC-positive group (*p* < 0.001, *p* = 0.001, *p* = 0.009, and *p* = 0.006, respectively; Figure 3E–H) (Table 3). Both Cr/IDC-positive and Cr/IDC-negative tumors exhibited an increase in ADC from 0 to 33 Hz of similar magnitude (Figure 3I).

Because Gleason grade may also correlate with microstructural parameters, sensitivity analyses adjusting for Gleason grade were performed to assess the direct relationship between Cr/IDC pattern and t_d_-dMRI-derived metrics. After adjustment, the differences remained significant: Cr/IDC-positive tumors exhibited significantly higher f_in_ and cellularity (*p* = 0.009 and *p* = 0.022, respectively) and lower ADC_0Hz_ (*p* = 0.007), ADC_DWI_ (*p* = 0.015).

Univariate and multivariable logistic regression analyses were further performed to explore the independent predictor of Cr/IDC status. In univariate analyses, PSA, PSAD, PI-RADS score, f_in_, cellularity, ADC_0Hz_, ADC_17Hz_, ADC_33Hz_, and ADC_DWI_ were significantly associated with Cr/IDC-positive tumors (all *p* < 0.05). Multicollinearity analysis suggested potential collinearity between PSA and PSAD (VIF = 13.6 and 12.9, respectively), as well as between f_in_ and cellularity (VIF = 23.0 and 34.7, respectively). After comprehensive evaluation of collinearity and clinical interpretability, PSAD and f_in_ were retained for multivariable analysis. Among the multiple ADC measurements, ADC_DWI_ was included to allow direct comparison with conventional mpMRI metrics. Finally, PSAD, PI-RADS score, f_in_, and ADC_DWI_ were included in the multivariable logistic regression model. Nonetheless, none of the included variables reached statistical significance after adjustment, as shown in Supplementary Table S3.

### 3.4. Diagnostic Performance of t_d_-dMRI-Derived Parameters for Cr/IDC Morphology

**Figure 4:**
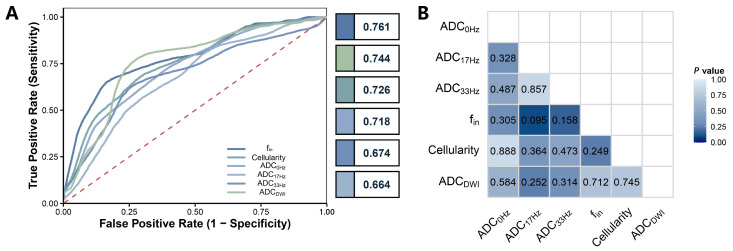
Diagnostic performance of the microstructural parameters for identifying Cr/IDC morphologies in PCa. (**A**) Receiver operating characteristic curves generated using 1000-sample bootstrap resampling; (**B**) Heatmap of *p*-values from pairwise DeLong tests comparing AUCs among different imaging parameters. Abbreviations: ADC_DWI_, ADC derived from diffusion-weighted imaging; ADC_0Hz_, diffusivity at 0 Hz; ADC_17Hz_, diffusivity at 17 Hz; ADC_33Hz_, diffusivity at 33 Hz; f_in_, intracellular volume fraction.

The diagnostic performance of microstructural parameters for identifying Cr/IDC morphologies in PCa is summarized in Table 4 and Figure 4. Among the microstructural metrics, f_in_ demonstrated the highest diagnostic performance for detecting Cr/IDC architecture (AUC = 0.757; 95% CI, 0.654–0.860), with a sensitivity of 69.4%, specificity of 81.8%, and accuracy of 73.7% at the exploratory optimal threshold determined by the Youden index. Its diagnostic performance remained stable in internal validation using 1000-sample bootstrap resampling, yielding a comparable bootstrap AUC of 0.761 (95% CI, 0.664–0.855) (Figure 4A). The diagnostic performance of f_in_ was slightly higher than that of the conventional ADC measurements, while the differences were not statistically significant (Figure 4B).

### 3.5. Interreader Reliability

Interobserver agreement for t_d_-dMRI-derived measurements was excellent, with intraclass correlation coefficients ranging from 0.899 to 0.995 (Table S4).

## 4. Discussion

Noninvasive identification of Cr/IDC morphologies in PCa is important for guiding management and predicting clinical outcomes. This study demonstrates that t_d_-dMRI-derived f_in_ and cellularity were significantly higher in Cr/IDC-positive tumors, whereas ADC values at 0, 17, and 33 Hz were lower than in Cr/IDC-negative tumors. These findings remained robust after adjustment for Gleason grade. Among the microstructural features, f_in_ demonstrated the highest diagnostic performance (AUC = 0.757; 95% CI, 0.654–0.860), suggesting its potential for identifying these aggressive histologic patterns.

Our analysis revealed that PCa with Cr/IDC morphologies exhibited higher pathologic grade. In addition, adverse pathologic features, including PNI and PSM, were more frequently observed in Cr/IDC-positive tumors. These findings are consistent with prior reports identifying Cr/IDC architecture as a pathologic hallmark of adverse clinical outcomes [27,28]. For risk stratification, Cr/IDC-positive tumors were more frequently classified into the high-risk group, highlighting the clinical importance of early identification of these aggressive histologic patterns.

Previous studies reported lower ADC values in tumors with Cr/IDC patterns [20,29,30]. In our study, ADC values at 0, 17, 33 Hz and ADC derived from DWI were also lower in Cr/IDC-positive tumors than in Cr/IDC-negative tumors. The difference in ADC_0Hz_ and ADC_DWI_ between the two groups remained significant after adjustment for Gleason grade, a well-established marker of tumor aggressiveness. This result may reflect increased tissue cellularity in IDC and compact arrangement of malignant epithelial cells within cribriform structures, which reduce intervening stroma and vasculature and thereby restrict water diffusion [29,31]. Additionally, Wu et al. reported differences in diffusion-time dependence among ISUP grade groups [15]; however, we did not observe such differences between Cr/IDC-positive and Cr/IDC-negative tumors. These observations suggest that diffusion-time dependence of water mobility may be more strongly influenced by pathologic grade than by Cr/IDC architecture. Nonetheless, validation in larger cohorts is warranted. Despite statistically significant differences in ADC values between Cr/IDC-positive and Cr/IDC-negative tumors, ADC primarily serves as a macroscopic indicator of water diffusivity and does not directly reflect microstructural properties at the cellular level.

Various imaging approaches have been developed to probe prostate tissue microstructure, including luminal water imaging [32], VERDICT (vascular, extracellular, and restricted diffusion for cytometry in tumors) [33], hybrid multidimensional MRI [34], and diffusion-relaxation spectrum imaging [11]. However, their clinical translation may be limited by complex acquisition requirements, prolonged scan times, or indirect assessment of cellular architecture. In contrast, by integrating q-space and t-space diffusion information within a time-dependent diffusion MRI framework, IMPULSED uses an analytical biophysical model to estimate interpretable microstructural parameters, providing information beyond conventional ADC measurement. Moreover, IMPULSED can be implemented on clinical MRI scanners, supporting its clinical translational value [15]. Its feasibility has been validated against histopathology in several tumor types, including glioma, breast tumor, ovarian cancer, etc. [35,36,37]. In PCa, previous studies have confirmed the potential of t_d_-dMRI to depict microstructural characteristics across different ISUP grade groups [12]. Wu et al. further reported that t_d_-dMRI showed superior accuracy for discriminating clinically significant PCa from clinically insignificant disease [15]. We extended this technique to map the microstructure of Cr/IDC patterns in PCa and found that f_in_ and cellularity were higher in Cr/IDC-positive tumors than in Cr/IDC-negative tumors. These results remained significant after adjustment for Gleason grade, suggesting that these parameters may not only reflect tumor pathologic grading but also capture microstructural features related to Cr/IDC patterns. From a biological perspective, this finding provides supporting evidence for the pathological relevance of IMPULSED-derived parameters. Elevated f_in_ and cellularity in Cr/IDC-positive tumors likely reflect the dense, compact growth patterns of these subtypes. Pathologically, Cr/IDC lesions are characterized by increased cellular density, reduced extracellular space, and higher architectural complexity, which may restrict water diffusion and enhance intracellular signal contribution [38].

The t_d_-dMRI-derived parameters, particularly f_in_, showed favorable diagnostic performance for identifying Cr/IDC morphologies, with an AUC of 0.757. However, its independent incremental value beyond clinical and conventional mpMRI parameters was not confirmed in multivariable analysis. This may be related to the joint analysis of Cr and IDC despite their distinct histologic architectures, which may introduce biological heterogeneity. In addition, the absence of quantitative information on Cr/IDC extent precluded assessment of potential dose–response effects, and lesion-level averaging within ROIs may have further diluted spatially heterogeneous tumor components, reducing sensitivity in multivariable models. Therefore, validation with more refined pathological stratification is warranted.

Several limitations should be noted. First, this was a single-center, retrospective study with a modest sample size, including only radical prostatectomy patients to ensure comprehensive histopathologic reference [39], which may limit generalizability to patients managed with other strategies. Validation in larger, multicenter cohorts is needed. Second, spatial co-registration between MRI-based ROIs and histopathology may be imperfect, as whole-mount sections were not available for all cases, which may introduce measurement uncertainty in t_d_-dMRI parameters. Third, Cr and IDC were analyzed together due to their frequent coexistence and shared prognostic implications. Separate analyses and quantitative assessment of Cr/IDC component burden within tumors were not feasible due to limited subgroup size and the lack of routine immunohistochemical confirmation, which represents an important direction for future research. Fourth, lesions smaller than 1 cm were excluded because of the current spatial resolution limitations of t_d_-dMRI, which may introduce selection bias. Finally, this study primarily focused on the feasibility of t_d_-dMRI for preoperative characterization of Cr/IDC, and further work is required to investigate its prognostic implications in long-term outcomes.

## 5. Conclusions

T_d_-dMRI-derived microstructural parameters show promise for characterizing Cr/IDC-related tissue alterations, while further validation in larger, multicenter cohorts is warranted.

## Figures and Tables

**Table 1:** Baseline participant and tumor characteristics.

Variable	Value
Age (years)	68.00 (62.00, 72.00)
PSA (ng/mL)	12.00 (8.20, 22.04)
PSAD (ng/mL^2^)	0.35 (0.20, 0.61)
Prostate volume (cm^3^)	33.58 (28.01, 48.30)
Tumor Diameter (cm)	1.80 (1.30, 2.40)
Location	
Peripheral zone	47 (49.5)
Transition zone	38 (40.0)
Both	10 (10.5)
PI-RADS score	
PI-RADS 3	14 (14.7)
PI-RADS 4	29 (30.5)
PI-RADS 5	52 (54.7)
ISUP grade group	
Grade group 1	11 (11.6)
Grade group 2	33 (34.7)
Grade group 3	26 (27.4)
Grade group 4	9 (9.5)
Grade group 5	16 (16.8)
Cr/IDC+	62 (65.3)
Cr/IDC−	33 (34.7)

Note: Data are reported as medians with interquartile ranges in parentheses or patient number with percentage in parentheses. Abbreviations: Cr/IDC, cribriform/intraductal carcinoma histologic pattern; ISUP, International Society of Urological Pathology; PI-RADS, Prostate Imaging and Reporting Data System; PSA, prostate-specific antigen; PSAD, prostate-specific antigen density.

**Table 2:** Comparison of pathologic characteristics between Cr/IDC-positive and Cr/IDC-negative prostate cancer.

Variable	Cr/IDC+ (*n* = 62)	Cr/IDC− (*n* = 33)	χ^2^	*p* Value
ISUP grade group			25.646	<0.001 *
Grade group 1	1 (1.6)	10 (30.3)		
Grade group 2	18 (29.0)	15 (45.5)		
Grade group 3	21 (33.9)	5 (15.2)		
Grade group 4	9 (14.5)	0		
Grade group 5	13 (21.0)	3 (9.1)		
Extraprostatic extension	30 (48.4)	11 (33.3)	1.989	0.158
Seminal vesicle invasion	14 (22.6)	4 (12.1)	1.534	0.215
Perineural invasion	54 (87.1)	23 (69.7)	4.246	0.039 *
Lymphovascular invasion	11 (17.7)	2 (6.1)	1.597	0.206
Positive surgical margin	35 (56.5)	9 (27.3)	7.375	0.007 *
D’Amico risk stratification			18.274	<0.001 *
Low risk	0	6 (18.2)		
Intermediate risk	16 (25.8)	15 (45.5)		
High risk	46 (74.2)	12 (36.4)		

Note: Data are expressed as patient number with percentage in parentheses. *p*-values were evaluated with the Pearson chi-square test or Fisher’s exact test. * *p* < 0.05. Abbreviations: Cr/IDC, cribriform/intraductal carcinoma histologic pattern.

**Table 3:** Comparison of time-dependent diffusion magnetic resonance imaging-derived microstructural parameters between Cr/IDC-positive and Cr/IDC-negative prostate cancer.

Variable	Cr/IDC+ (*n* = 62)	Cr/IDC− (*n* = 33)	*t/z* Value	*p* Value	*p* Value Adjusted for Gleason Grade
D_ex_ (μm^2^/ms)	2.13 (2.00, 2.45)	2.14 (2.00, 2.31)	0.485 ^b^	0.628	0.519
d (μm)	18.81 (16.52, 21.01)	20.13 (17.75, 21.59)	−0.774 ^b^	0.439	0.701
f_in_	0.40 (0.31, 0.45)	0.28 (0.17, 0.33)	0.411 ^b^	<0.001 *	0.009 *
Cellularity (μm^−1^)	2.06 ± 0.72	1.53 ± 0.69	−3.513 ^a^	<0.001 *	0.022 *
ADC_0Hz_ (μm^2^/ms)	1.13 (0.99, 1.45)	1.50 (1.18, 1.75)	−3.433 ^b^	0.001 *	0.007 *
ADC_17Hz_ (μm^2^/ms)	1.40 (1.20, 1.65)	1.65 (1.38, 1.93)	−2.618 ^b^	0.009 *	0.074
ADC_33Hz_ (μm^2^/ms)	1.50 (1.30, 1.80)	1.75 (1.50, 1.93)	−2.749 ^b^	0.006 *	0.080
ADC_DWI_ (μm^2^/ms)	0.80 (0.73, 0.85)	0.91 (0.85, 1.06)	−3.828 ^b^	<0.001 *	0.015 *

Note: Data are means ± SDs or median (IQR). ^a^ Student’s *t*-test. ^b^ Mann–Whitney U test. Adjusted *p*-values were obtained from Gleason grade-adjusted multivariable linear regression models. * *p* < 0.05. Abbreviations: ADC_0Hz_, diffusivity at 0 Hz; ADC_17Hz_, diffusivity at 17 Hz; ADC_33Hz_, diffusivity at 33 Hz; ADC_DWI_, ADC derived from diffusion-weighted imaging; Cr/IDC, cribriform/intraductal carcinoma histologic pattern; D_ex_, extracellular diffusivity; d, cell diameter; f_in_, intracellular volume fraction.

**Table 4:** Diagnostic performance of time-dependent diffusion magnetic resonance imaging-derived microstructural parameters for identifying Cr/IDC morphologies in prostate cancer.

Parameter	AUC (95% CI)	Cutoff Value	Sensitivity (%)	Specificity (%)	Accuracy (%)
f_in_	0.757 (0.654–0.860)	0.344	69.4 (56.3–80.4)	81.8 (64.5–93.0)	73.7 (63.6–82.2)
Cellularity (μm^−1^)	0.721 (0.611–0.832)	1.798	67.7(54.7–79.1)	72.7 (54.5–86.7)	69.5 (59.2–78.5)
ADC_0Hz_ (μm^2^/ms)	0.714 (0.604–0.825)	1.275	66.1 (53.0––77.7)	66.7 (48.2–82.0)	66.3 (55.9–75.7)
ADC_17Hz_ (μm^2^/ms)	0.663 (0.545–0.782)	1.475	59.7 (46.4–71.9)	69.7 (51.3–84.4)	63.2 (52.6–72.8)
ADC_33Hz_ (μm^2^/ms)	0.672 (0.557–0.786)	1.525	62.9(49.7–74.8)	75.8(57.7–88.9)	67.4 (57.0–76.6)
ADC_DWI_ (μm^2^/ms)	0.739 (0.625–0.853)	0.875	82.3 (70.5–90.8)	69.7 (51.3–84.4)	77.9 (68.2–85.8)

Note: Data are mean values, with bootstrapped 95% confidence intervals in parentheses. Abbreviations: ADC_0Hz_, diffusivity at 0 Hz; ADC_17Hz_, diffusivity at 17 Hz; ADC_33Hz_, diffusivity at 33 Hz; ADC_DWI_, ADC derived from diffusion-weighted imaging; AUC, area under the receiver operating characteristic curve; Cr/IDC, cribriform/intraductal carcinoma histologic pattern; f_in_, intracellular volume fraction.

## Data Availability

The data presented in this study are available from the corresponding author upon reasonable request.

## Supplementary Materials

The following supporting information can be downloaded at https://www.mdpi.com/article/10.3390/cancers18132056/s1: Table S1: Sequence parameters for diagnostic MRI; Table S2: Sequence parameters for diagnostic MRI; Table S3: Univariate and Multivariable Logistic Regression Analyses for Identifying Cr/IDC-Positive Prostate Cancer. Table S4: Results of two-reader reliability analysis.

## Abbreviations

PCa	Prostate cancer
Cr	Cribriform
IDC	Intraductal carcinoma
t_d_-dMRI	time-dependent diffusion MRI
IMPULSED	Imaging Microstructural Parameters Using Limited Spectrally Edited Diffusion
OGSE	Oscillating gradient spin-echo
PGSE	Pulsed gradient spin-echo
DWI	Diffusion weighted imaging
ADC	Apparent diffusion coefficient
D_ex_	extracellular diffusivity
f_in_	Intracellular volume fraction
