# Supplementary material for: Time-Dependent Diffusion MRI-Based Microstructural Mapping for Characterization of Cribriform and Intraductal Carcinoma Morphologies in Prostate Cancer: A Preliminary Study
Source: Cancers (Basel). 2026 Jun 25;18(13):2056. doi: 10.3390/cancers18132056 (PMC13359592; doi:10.3390/cancers18132056)
Supplement: Supplementary file 1 [file cancers-18-02056-s001.zip › cancers-4357664-supplementary.pdf]

Table S1: Sequence parameters for diagnostic MRI

Parameters	T1WI	T2WI	DWI	DCE-MRI
sequence	Fast spin echo	Fast spin echo	EPI(MUSE)	GRE-DISCO
Imaging plane	axial	axial, coronal, sagittal	axial	o-axial
Repetition time/ Echo time (ms)	502/8.9	5248/160, 4366/100, 4326/120	2500/62.2	4.2/1.9
Flip angle(degrees)	111	111,111,111	/	15
Field of view(cm^2)	25×32.5	20×20,22×22,20×20	25×25	24×24
Explannar Resolution(mm^2)	0.6×0.8	0.8×0.8,0.6×0.6,0.6×0.8	1.6×1.6	1.1×1.1
Number of slices	19	19,18,19	19	50
Slice thickness(mm)	3.0	3.0,3.0,3.0	3.0	1.6
Matrix	416×390	320×260，352×352，352×260	160×160	220×220
Number of excitations	1.5	2, 2.5, 2.0	2.0,10,11	1
Echo train length	4	28, 27, 24	/	/
Bandwidth(kHz)	50.00	35.71, 62.5, 41.67	250	50
B value (s/mm^2)	/	/	50,1000,1500	/
Acceleration factor	1.75	1.21, 2.0, 1.5	1.0	1.8
Acquisition time(min:s)	1:44	2:11, 2:11, 1:48	3:05, 3:24	2:40

Abbreviations: T1WI, T1 weighted imaging; T2WI, T2 weighted imaging; DWI, diffusion weighted imaging; DCE-MRI, Dynamic contrast-enhanced MRI

Table S2: Sequence parameters for the time-dependent diffusion MRI protocols

Sequence	Big-Delta (ms)	Small-Delta (ms)	Cycle	f [Hz]	Effective diffusion time [ms]	b value [s/mm^2]	Diffusion directions	Scan time
OGSE	70	60.7	2	33	7.6	300,600	6	1:38
OGSE	70	59	1	17	14.8	200, 400, 800, 1200	6	3:02
PGSE	70	8	/	0	67.3	200, 400, 800, 1200	6	3:02

Abbreviations: OGSE, oscillating gradient spin-echo; PGSE, pulsed gradient spin-echo

Table S3: Univariate and multivariate logistic regression analyses for identifying Cr/IDC-positive prostate cancer

Variables	Univariate Analyses			VIF	Multivariate Analyses		
	OR	95% CI	P-value		OR	95% CI	P-value
Age (years)	1.026	0.961-1.096	0.441	1.34			
PSA (ng/mL)	1.046	1.009-1.098	0.037*	13.60			
PSAD (ng/mL^2)	15.293	2.722-151.131	0.008*	12.90	7.006	1.287-76.234	0.066
Prostate volume (cm^3)	0.975	0.949-1.000	0.057	2.56			
Tumor Diameter (cm)	1.090	0.679-1.812	0.728	2.08			
PI-RADS score							
PI-RADS 3	/						
PI-RADS 4	4.000	1.077-16.488	0.044*	1.42	1.736	0.339-9.209	0.506
PI-RADS 5	4.440	1.316-16.594	0.019*	1.42	0.822	0.157-4.114	0.811
D_{ex} (μm^2/ms)	1.865	0.546-6.944	0.331	1.52			
d (μm)	0.981	0.863-1.111	0.763	10.50			
f_{in}	5261.934	74.719-631474.482	<0.001*	23.00	299.932	0.605-241506.966	0.080
Cellularity (μm^{-1})	3.169	1.619-6.833	0.002*	34.70			
ADC_{0Hz} (μm^2/ms)	0.103	0.023-0.378	0.001*	2.72			
ADC_{17Hz} (μm^2/ms)	0.220	0.061-0.713	0.014*	3.02			
ADC_{33Hz} (μm^2/ms)	0.317	0.098-0.936	0.044*	2.13			
ADC_{DWI}(μm2/ms)	0.005	0.000-0.094	<0.001*	2.48	0.103	0.001-5.531	0.274

*P < 0.05. Abbreviations: Cr/IDC, cribriform/intraductal carcinoma histologic pattern; VIF, variance inflation factors; PI-RADS, Prostate Imaging and Reporting Data System; PSA, prostate-specific antigen; PSAD, prostate-specific antigen density; D_{ex}, extracellular diffusivity; d, cell diameter; f_{in}, intracellular volume fraction; ADC_{0Hz}, diffusivity at 0 Hz; ADC_{17Hz}, diffusivity at 17 Hz; ADC_{33Hz}, diffusivity at 33 Hz; ADC_{DWI}, ADC derived from diffusion-weighted imaging.

Table S4: Results of two-reader reliability analysis

Parameters	ICC	95%CI
D_{ex}	0.986	0.979, 0.991
d	0.986	0.98, 0.991
f_{in}	0.989	0.983, 0.992
Cellularity	0.986	0.979, 0.991
ADC_{33Hz}	0.995	0.993, 0.997
ADC_{17Hz}	0.986	0.979, 0.991
ADC_{0Hz}	0.974	0.960, 0.982
ADC_{DWI}	0.996	0.993, 0.997

Note: Reliability was considered poor (ICC <0.40), moderate (0.40 ≤ ICC <0.60), good (0.60 ≤ ICC <0.80), or excellent (ICC ≥ 0.80). Abbreviations: ICC: intraclass correlation coefficient; D_{ex}, extracellular diffusivity; d, cell diameter; $f_{in,}$ intracellular volume fraction; $ADC_{0Hz,}$ diffusivity at 0 Hz, ADC_{17Hz} , diffusivity at 17 Hz, ADC_{33Hz}, diffusivity at 33 Hz; ADC_{DWI}, ADC derived from diffusion-weighted imaging.
